# “Green” Aqueous Synthesis and Advanced Spectral Characterization of Size-Selected Cu_2_ZnSnS_4_ Nanocrystal Inks

**DOI:** 10.1038/s41598-018-32004-1

**Published:** 2018-09-12

**Authors:** Oleksandr Stroyuk, Alexandra Raevskaya, Oleksandr Selyshchev, Volodymyr Dzhagan, Nikolai Gaponik, Dietrich R. T. Zahn, Alexander Eychmüller

**Affiliations:** 1 Physical Chemistry, TU Dresden, 01062 Dresden, Germany; 2 L.V. Pysarzhevsky Institute of Physical Chemistry, National Academy of Sciences of Ukraine, Kyiv, 03028 Ukraine; 3 Semiconductor Physics, Chemnitz University of Technology, 09107 Chemnitz, Germany; 4 V. E. Lashkaryov Institute of Semiconductors Physics, National Academy of Sciences of Ukraine, Kyiv, 03028 Ukraine

## Abstract

Structure, composition, and optical properties of colloidal mercaptoacetate-stabilized Cu_2_ZnSnS_4_ (CZTS) nanocrystal inks produced by a “green” method directly in aqueous solutions were characterized. A size-selective precipitation procedure using 2-propanol as a non-solvent allows separating a series of fractions of CZTS nanocrystals with an average size (bandgap) varying from 3 nm (1.72 eV) to 2 nm (2.04 eV). The size-selected CZTS nanocrystals revealed also phonon confinement, with the main phonon mode frequency varying by about 4 cm^−1^ between 2 nm and 3 nm NCs.

## Introduction

Multinary metal-chalcogenide nanocrystals (NCs), in particular, ternary CuInS_2_ and AgInS_2_ as well as quaternary Cu_2_ZnSnS_4_ (CZTS) and Cu_2_ZnSnSe_4_ (CZTSe) NCs show currently great promise for various light-harvesting applications including photovoltaics and photocatalysis^1–11^. The kesterite CZTS and CZTSe compounds formed by widely abundant constituents combine high absorption coefficients and light sensitivity through the visible and near IR spectral ranges with a broad variability of properties due to large tolerances to non-stoichiometry, the substitution of Zn with other cations (Ba, Co, Fe, Ni, Mn, *etc*.), a partial substitution of Sn with Ge as well as the alloying of sulfur and selenium in a single chalcogenide sublattice^2–4,6,7,10–12^.

Recent reports also showed that optical and photophysical properties of CZTS NCs can be tuned by changing the NC size due to spatial exciton confinement that can be observed for CZTS NCs smaller than ~5 nm^4,6,10,13^. Nanocrystalline CZTS and CZTSe typically reveal a much higher photocatalytic activity as compared to their bulk counterparts and can be easily introduced into photocathodes and counter electrodes of solar cells^2,4,6–10^. Simultaneously, the trend to utilize concentrated CZTS NC inks for the solution-based production of microcrystalline CZTS/CZTSe solar cell absorbers becomes ever more pronounced in the last years^3–5,10,14^. All these factors stimulated the development of new synthetic approaches allowing CZTS NCs with a defined size, lattice type, shape, and composition to be produced^6,7,10,11,15,16^.

Typically, CZTS NCs are produced by well-reported heating up/hot injection syntheses performed in high-boiling point organic solvents (such as oleylamine or octadecene), where a precise control over the NC size, phase, and shape can be achieved^17–33^. Nanocrystalline CZTS films with a pre-defined composition can be produced by the thermolysis of a single precursor containing all four components^34–36^. For photocatalytic applications the nanocrystalline CZTS is typically synthesized by various hydrothermal/solvothermal approaches^13,37–48^ and by microwave-assisted methods^49–52^. The former methods need to be coupled to post-syntheses treatments, in particular aimed at the phase-transfer of CZTS NCs into water and other polar solvents, while the latter protocols yield mostly powdered samples which have to be converted into dispersions/suspensions for the preparation of solar cell absorbers or photoelectrodes. In this view, the development of more straightforward synthetic approaches, for example, those of synthesizing CZTS NCs directly in water are of considerable importance. However, the examples of aqueous syntheses of CZTS NCs are quite rare^53,54^ typically yielding strongly agglomerated NCs and providing no reliable means for the control of the NC size and size distribution. Only recently aqueous syntheses with Sn_2_S_6_^4−^ complex anions as capping ligands were reported to produce stable and uniform CZTS NCs^55,56^.

In the present paper, we report on an aqueous synthesis of colloidal CZTS NCs stabilized by mercaptoacetate (MA) anions that can be subjected to post-synthesis size-selective precipitation. This method allows for the separation of 8–9 fractions of size-selected CZTS NCs from a starting ensemble with an average NC size varying in the fractions between 2 and 3 nm. The size-dependence of the electronic properties of CZTS is especially strong in this size range and the availability of multiple NC fractions allows the influence of the spatial exciton confinement on the optical properties of CZTS NCs, in particular on the absorption band edge position, to be reliably monitored. Besides, the size-dependence of the phonon spectra was probed by resonance Raman spectroscopy. Unlike other reports, where the CZTS NCs of different sizes were prepared by varying the temperature or duration of synthesis, both factors inevitably affecting the NC crystallinity and chemical composition, we can selectively probe the size dependences of the NC properties separated from other possible influences.

## Materials and Methods

Mercaptoacetic acid (MAA), Cu(NO_3_)_2_ × 3H_2_O, SnCl_2_ × 2H_2_O, SnCl_4_ × 5H_2_O, Zn(CH_3_COO)_2_ × 2H_2_O, NaOH,  Na_2_S × 9H_2_O, 2-propanol were supplied by Sigma Aldrich and Acros Organics and used without any further purification.

### Preparation of stock tin precursor solution

A stock aqueous 0.5 M solution of SnCl_2_ in 4.0 M NaOH was prepared by slowly pouring an aqueous 1.0 M suspension of SnCl_2_ × 2H_2_O into a hot (70–80 °C) aqueous 8.0 M solution of NaOH (volumic ratio of the suspension and NaOH solutions were 1:1). The suspension was produced by mixing SnCl_2_ crystal hydrate with water to obtain 1.0 M SnCl_2_. This procedure allows the formation of a black tin hydroxide/oxide precipitate to be avoided and water-soluble sodium hydrostannate to be obtained. The addition of Na_2_S to fresh alkaline SnCl_2_ solution yields brown SnS precipitate, however, after a storage under ambient air Sn^2+^ is completely oxidized to Sn^4+^ and the Na_2_S probe results in the deposition of yellow SnS_2_ precipitate that dissolves in a Na_2_S excess, contrary to SnS. We tried both fresh and stored stock tin chloride solutions and found that the properties of the final CZTS NCs do not depend on the oxidation state of tin in the stock solution. Both SnCl_2_ and SnCl_4_ compounds can be used for the synthesis yielding essentially the same results.

### Preparation of colloidal CZTS NCs

Colloidal CZTS NCs were synthesized in a reaction between a mixture of MA complexes of Cu^2+^, Zn^2+^, and Sn^2+^ and sodium sulfide in water. In a typical synthesis, 0.3 mL aqueous 1.0 M Cu(NO_3_)_2_ solution, 0.3 mL aqueous 0.5 M SnCl_2_ or SnCl_4_ (both with 4.0 M NaOH) solution, and 0.15 mL aqueous 1.0 M Zn(CH_3_COO)_2_ solution were consecutively added to 5.5 mL deionized water (DI water) under stirring followed by the addition of 3.0 mL aqueous 1.0 M MAA solution and 0.32 mL aqueous 1.0 M NaOH solution. Finally, 0.3 mL aqueous 1.0 M Na_2_S solution was added and the resulting mixture kept at 95–98 °C for 10 min. In different series of experiments, we varied the contents of MAA, CZTS concentration (with a constant ratio of the components), and the duration of the final heating, while keeping other parameters constant.

### Size selection of CZTS NCs

The size-selected CZTS NCs were produced from the above-described colloidal solution by a selective precipitation method similar to the one in our previous reports on Ag-In-S NCs^57–59^. In a typical approach, to 10.0 mL colloidal CZTS solution 0.5 mL of 2-propanol (as a non-solvent) was added and the solution was subjected to the centrifugation at 10000 rpm for 2 min. The non-solvent addition and centrifugation resulted in a partial precipitation of colloidal NCs that were separated by the decantation of the supernatant solution and re-dispersed in 0.5 mL DI water resulting in a solution named “fraction No. 1”. This procedure was repeated 4 times with the supernatant solutions and 0.5 mL 2-propanol, each iteration resulting in fractions No. 2–5. The next two fractions No. 6 and 7 were produced by adding 1.0 mL 2-propanol to the corresponding supernatant solutions. The last two fractions No. 8 and 9 were produced by adding 2.0 mL and 3.0 mL 2-propanol, respectively. The total amount of added 2-propanol was 9.5 mL.

### Instrumentation

The absorption spectra were recorded using a UV-vis spectrophotometer Cary 60 in standard 1.0 cm optical quartz cuvettes. X-ray Photoelectron Spectroscopy (XPS) studies were performed using an ESCALAB™ 250Xi X-ray Photoelectron Spectrometer Microprobe (Thermo Scientific). For XPS measurements the colloids were drop-casted on Au/Si substrate and dried under vacuum. The spectra were acquired under excitation of a 650 µm spot with a monochromized Al *K*_α_ (*hν* = 1486.6 eV) X-ray source and a pass energy of 20 eV (spectral resolution of 0.5 eV) for core-level spectra, of 40 eV (resolution of 0.9 eV) for Auger spectra, and of 200 eV for survey spectra (resolution of ~4 eV). During the XPS measurement, the sample was flooded with low kinetic energy (~0.1 eV) electrons to prevent possible charging. Spectra deconvolution and quantification were performed using the Thermo Scientific^TM^ Avantage Software. Raman spectra were excited with a 514.7 nm DPSS laser (Cobolt) and registered with a LabRam HR spectrometer with a spectral resolution of 5 cm^−1^. The peak positions were determined with an accuracy ~1 cm^−1^. The incident laser power was kept below 0.1 mW in order to avoid sample heating under the microscope objective (50×).

X-ray diffractograms were recorded using a Bruker D2 Phaser diffractometer in an angle range of 2Θ = 10–100° with a rate of 0.05°/min with monochromatized copper *K*_α_ irradiation. The samples were prepared by dropcasting colloidal NC solutions mixed with acetone (1:1) on a silicon wafer (which was used as an internal standard for the evaluation of diffraction peak widths) followed by drying in a stream of nitrogen at room temperature. Transmission electron microscopy (TEM) was performed using a FEI Tecnai G2 microscope at an accelerating voltage of 300 kV. SEM and EDX were acquired using a Nova NanoSEM scanning electron microscope equipped with an EDX Bruker AXS Microanalysis setup.

## Results and Discussion

The interaction between a mixture of Cu(I), Zn(II), and Sn(IV) mercaptoacetate complexes with sodium sulfide at temperatures close to the water boiling point results in the formation of dark brown colloidal CZTS solutions stable when exposed to the ambience. The mercapto-groups of MAA form covalent bonds with under-coordinated metal ions on the NC surface thus retarting the NC growth. At the same time, the carboxyl group of MAA is deprotonated in alkaline solutions thus creating an electrostatic barrier arround each particle preventing the NC agglomeration and imparting the colloidal CZTS solutions with a prolonged stability.

**Figure 1:**
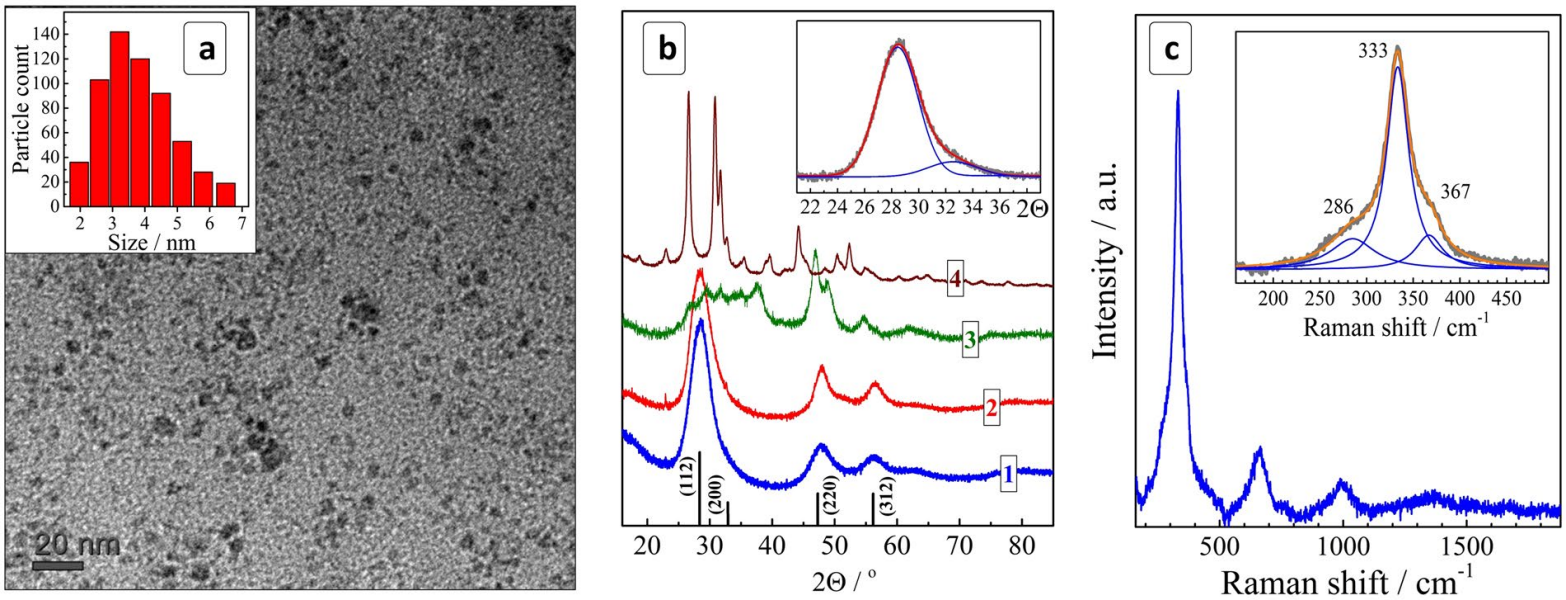
(**a**) TEM image and size distribution of CZTS NCs. (**b**) X-ray diffractograms of CZTS NCs (curve 1) as well as Cu-Sn-S (2), Cu-S (3), and Sn-S (4) samples produced under the same conditions. Black vertical lines show the positions of bulk kesterite diffraction peaks with corresponding face indices (JCPDS No. 26-0575). Inset: fitting of the (112/200) peak range with two Gauss profiles. (**c**) Raman spectrum of unfractionated CZTS NCs. Insert: fitting of a section of the first-order phonon range with a set of three Lorentz profiles.

The colloidal NCs can be precipitated with 2-propanol, separated from the maternal solution, and redispersed again in DI water (colloids with a lower stability in ambient air) or a sodium mercaptoacetate solution. An excess of sodium MA introduced during the redispersion increases the stability of the colloids that can be stored for months without appreciable precipitations or changes of the optical properties. TEM showed that such colloids contain 2–7 nm particles with a size distribution centered around 3–4 nm (Fig. 1a). The stability and absorption spectra of colloidal CZTS solutions were found to be almost unaffected by large variations of the heating duration (1–60 min), the concentration of CZTS precursors (0.006–0.075 M in terms of a CZTS unit) as well as by the presence of an excess of MA anions (Electronic Supplementary Information (ESI), Fig. S1). The concentration of 0.075 M corresponds to a Cu_2_ZnSnS_4_ content of ~33 g/L.

In the following discussion we present characterizations of the structure and composition as well as some optical properties of CZTS NCs synthesized at a nominal molar Cu:Zn:Sn ratio of 2:1:1, and report the characteristics of the size-selected CZTS NCs produced from initial colloidal CZTS NC ensembles by the size-sensitive fractional precipitation.

The X-ray diffraction pattern of CZTS NCs reveal a distinct tetragonal kesterite motif with the most prominent characteristic and broadened reflections found at 28.4°, 47.8°, and 56.2° (Fig. 1b, curve 1)^18,19,39,60,61^. The average size of CZTS NCs estimated from the full width at half-maximum (FWHM) of the most intense (110) peak (Fig. 1b, insert) was found to be ~3 nm, in agreement with the TEM results. The XRD allows us to exclude the presence of possible Cu-S and Sn-S phases that have distinctly different diffraction patterns (Fig. 1b, curves 3 and 4). The Cu-Sn-S phase reveals an XRD pattern very close to that of kesterite NCs (Fig. 1b, curve 2) and, therefore, a conclusion on the presence/absence of this phase cannot be drawn exclusively from the XRD data.

Raman scattering from the CZTS NCs deposited on a glass substrate and dried in vacuum was probed under resonant excitation at λ_exc_ = 514.7 nm. The NCs revealed a sharp first-order phonon peak of CZTS at about 333 cm^−1^ and a series of higher-order phonon peaks (Fig. 1c) revealing the high crystallinity of the synthesized NCs^62^. The first-order spectrum could be fitted with a combination of three Lorentz profiles showing maxima at 286 cm^−1^, 333 cm^−1^, and 367 cm^−1^, all three frequencies being characteristic for kesterite CZTS compounds^12,18,24,38,39,60,63–66^. Similar to the case of X-ray diffraction, the Raman spectra of the obtained CZTS NCs differ strongly from the spectra of the Cu-S and Sn-S NC phases synthesized under the same conditions as the CZTS NCs (ESI, Fig. S2, curves 3,4), allowing us to exclude the formation of these compounds. Additionally, the Raman spectra of the Cu-Zn-Sn-S and Cu-Sn-S phases showed quite distinct differences in the peak positions and band shapes as well (compare curves 1 and 2 in Fig. S2), allowing also the presence of Cu-Sn-S to be definitively excluded as a major contributor in the studied CZTS samples. We found that the results of Raman measurements depend strongly on the preparation history of the samples for the optical measurements, in particular, on humidity, air presence, as well as on the type of substrate used (silicon, glass, ITO, *etc*.) and on the excitation intensity. The details of the Raman investigation are beyond the scope of the present paper and will be published elsewhere as a separate contribution.

To assess the composition of CZTS NCs and the valence states of the NC constituents we studied the unfractionated CZTS colloid containing an excess of sodium mercaptoacetate by X-ray photoelectron spectroscopy. A survey spectrum (ESI, Fig. S3) shows the presence of the elements Cu, Sn, Zn, and S as well as a ~15% admixture of sodium, carbon and oxygen from MA anions and contaminations from adventitious carbon and water, respectively.

**Figure 2:**
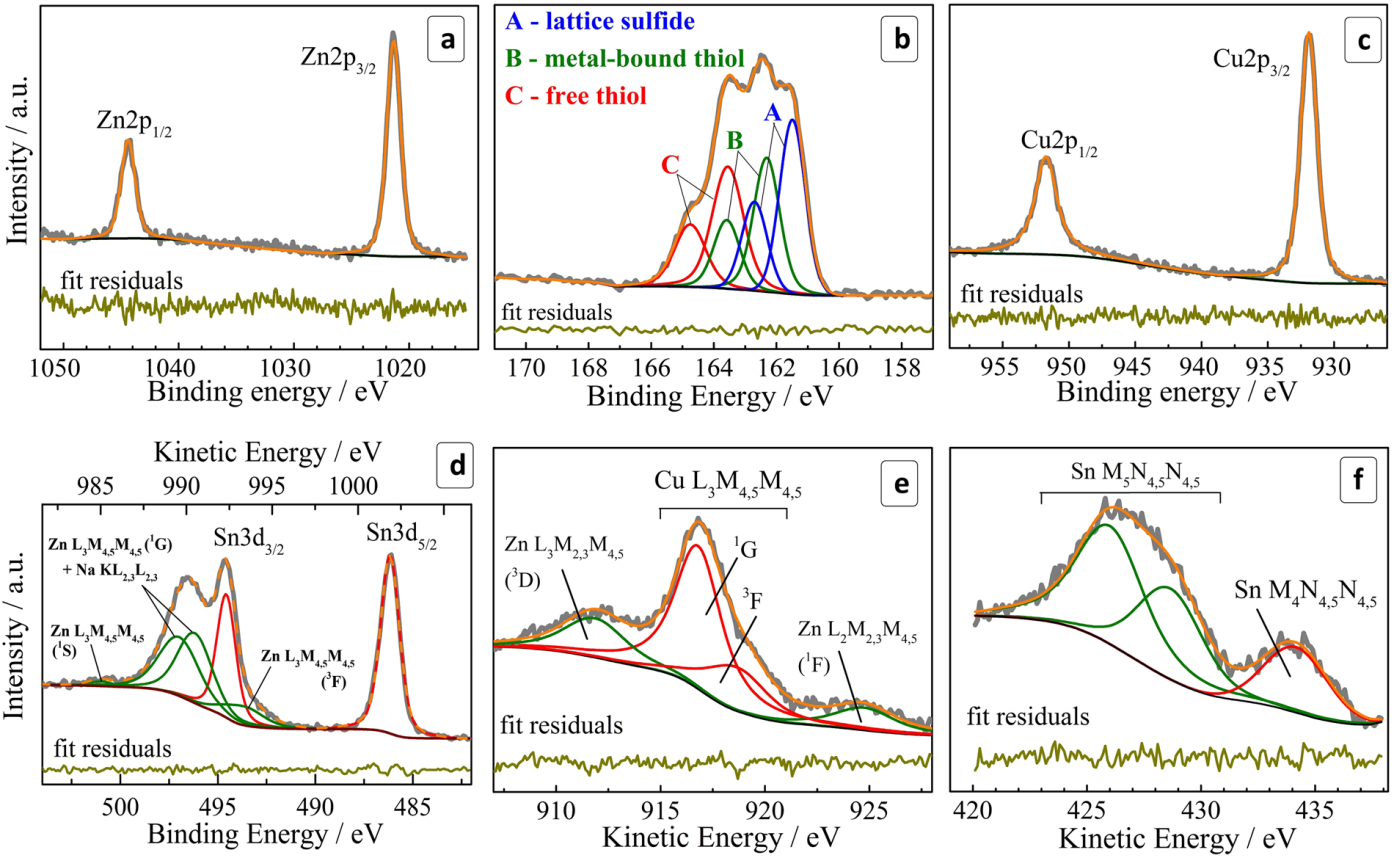
(**a**–**d**) High-resolution XPS spectra in the range of Zn 2p (**a**), S 2p (**b**), Cu 2p (**c**), and Sn 3d (**d**). (**e,f**) High-resolution spectra in the range of Auger electrons for copper (**e**) and tin (**f**). Attribution of Sn, Cu, Zn, and Na Auger peaks was performed according to refs^82–84^.

A high-resolution X-ray photoelectron spectrum in the range of Zn 2p electron binding energy (Fig. 2a) shows a characteristic doublet at 1021 eV and 1044 eV with a spin-orbit splitting of 23 eV typical for Zn^2+^ in chalcogenide lattices^24,43,47,61,67–69^. The high quality of the fit attests the absence of other forms of Zn(II) in the system under scrutiny.

The S 2p range (Fig. 2b) reveals a complex spectral structure that can be deconvoluted into three doublets, each with a spin-orbit splitting of 1.2 eV characteristic of S(II). The doublets at 161.5/163.7 eV (A), 162.3/163.5 eV (B), and 163.6/164.8 eV (C) were assigned respectively to sulfide anions in the CZTS lattice (A), thiol groups of MA anions bound to the metal atoms of the CZTS NC surface (B), and free thiol moieties (C)^70^. The latter contribution originates most probably from the sodium mercaptoacetate used to disperse NCs after the purification. No spectral features were observed at energies higher than 166 eV typical for S^0^ or SO_x_^70^, indicating that the sulfide sublattice of CZTS NCs is stable against oxidation.

The copper 2p range shows a doublet at 931.9/951.8 eV (Fig. 2c). Deconvolution reveals different FWHM for Cu2p_1/2_ and Cu2p_3/2_ components – 2.15 and 1.50 eV, respectively. This broadening is a result of an additional relaxation path for the L_2_ (2p_1/2_) hole due to L_2_L_3_M_4,5_ Coster-Cronig transition, which reduces the lifetime of the hole and broadens the Cu2p_1/2_ peak^71^. The positions of peaks, the distance between them (19.9 eV), the absence of an additional contribution at around 934/954 eV, as well as the absence of paramagnetic Cu(II) satellite peaks at ~940–945 eV^72^ indicate that copper is present in the sample solely as Cu(I) typically found in CZTS NCs^24,43,47,61,67–69,73^. Nevertheless, as the differences reported in the literature between the positions of Cu(I) and Cu(II) compounds (oxides and chalcogenides) are rather small, we examined additionally with high-resolution XPS spectra the range of the Auger electron peak of copper, which is more sensitive to the chemical surrounding than the XPS peaks. The spectrum of Cu L_3_M_4,5_M_4,5_ Auger electrons (Fig. 2e) shows the most intense Cu L_3_M_4,5_M_4,5_ (^1^G) peak at 916.8 eV (in the kinetic energy scale) as well as an Auger parameter of 1849 eV both typical for copper(I)^69,73^ thus supporting our above assignment of copper to the Cu(I) state.

The correct fit of the Sn 3d_3/2_ electron binding energy range requires the Auger peaks of Na KL_2,3_L_2,3_ and Zn L_3_M_4,5_M_4,5_ to be taken into account (Fig. 2d). The observed Sn 3d_5/2_ peak centered at 486.3 eV and the spin-orbit splitting of 8.5 eV are typical for Sn(IV)^24,43,47,61,67–69,73^. This assignment is supported by an analysis of the Sn M_4,5_N_4,5_N_4,5_ Auger electron energy spectrum (Fig. 2f) revealing the maximum of Sn M_4_N_4,5_N_4,5_ peak at 434 eV and an Auger parameter of 920 eV, both typical for Sn(IV) and reported for CZTS NCs produced by other methods^69^.

In summary, the high-resolution XPS results confirmed all four constituents of the CZTS NCs to be in the expected oxidation states – Cu(I), Zn(II), Sn(IV), and S(II). The X-ray photoelectron spectra of CZTS NCs synthesized starting from Sn(II)-MA and Sn(IV)-MA complexes were found to be identical further showing the formation of the same CZTS phase irrespective of the oxidation state of the Sn precursor. It should be noted that XPS studies of fractionated size-selected CZTS colloids (fractions # 1,6,8) showed the presence of only Cu(I), Zn(II) and Sn(IV), which is quite expected because no other forms of these elements were found in the original colloidal ensemble. In addition to the above-discussed elements we observed the presence of oxygen from mercaptoacetate ligands and adsorbed water as well as sodium ions the latter most probably compensating the charge of the carboxylate anions (ESI, Fig. S3).

The composition of the CZTS NCs revealed by XPS survey spectra was found to deviate quite strongly from the Cu_2_ZnSnS_4_ stoichiometry showing atomic Cu:Sn and Zn:Sn ratios of 5:1 and 3:1, respectively. At the same time, an EDX study of CZTS NCs deposited on ITO, collected from a 15 × 15 μm^2^ spot, showed a Cu:Zn:Sn ratio much closer to the kesterite stoichiometry (ESI, Table S1) than the ratio derived from the XPS results, as well as a uniform distribution of the elements over the film area (ESI, Fig. S4). The accuracy of EDX measurements is, however, compromised to some extent by an overlapping of the Sn signal from the sample with the In and Sn signals from the ITO support, which may result in an overestimation of the tin content and a large error (ESI, Table S1). At the same time, XPS is a surface-sensitive method and the strong deviation obtained of the compositions of the CZTS NCs from the stoichiometry can be an indication that the surface layer of NCs is enriched with copper and zinc. Indeed, it is quite reasonable to expect that the surface ligand shell is dominated by the Cu-MA and Zn-MA species, as the stability of mercaptoacetate complexes increases from tin to zinc to copper. Therefore, the combined EDX and XPS results can be considered as an indication of the NCs consisting of CZTS “cores” with a composition close to stoichiometry and an outer ligand “shell” enriched with copper and zinc bound to MA anions. Strong evidence of the CZTS phase being the main part of the NCs was provided by the phonon Raman spectra discussed above.

The separation of an ensemble of colloidal NCs into a series of fractions with different average sizes is typically performed by the addition of a non-solvent resulting in the precipitation of a portion of the largest NCs in the ensemble. This method is a powerful tool for producing and investigating a size-selected series of NCs having a common synthesis history and the same composition. Every NC in the original ensemble is formed under identical conditions and, therefore, the average size can be considered as the sole parameter varied in this process. This feature contrasts to the well-reported approach of size variation via changes in the heating temperature/duration in the heating up/hot injection syntheses, when not only the average NC size, but also the lattice perfection (density of defects), elemental composition, and surface chemistry of the NCs may be considerably affected.

Recently, we showed the feasibility of the size-selective fractionation of MA-capped^57,58^ and glutathione-capped^59^ aqueous Ag-In-S/ZnS NCs into a series of bright luminophors with different emission colors using 2-propanol as a non-solvent. Here, we extend this methodology to the MA-capped CZTS NCs. As described in the experimental section, the portion-wise addition of 2-propanol results in the precipitation of fractions of CZTS NCs that can be separated and redispersed in aqueous solutions of sodium mercaptoacetate thus yielding a series of 8–9 samples with different optical properties.

**Figure 3:**
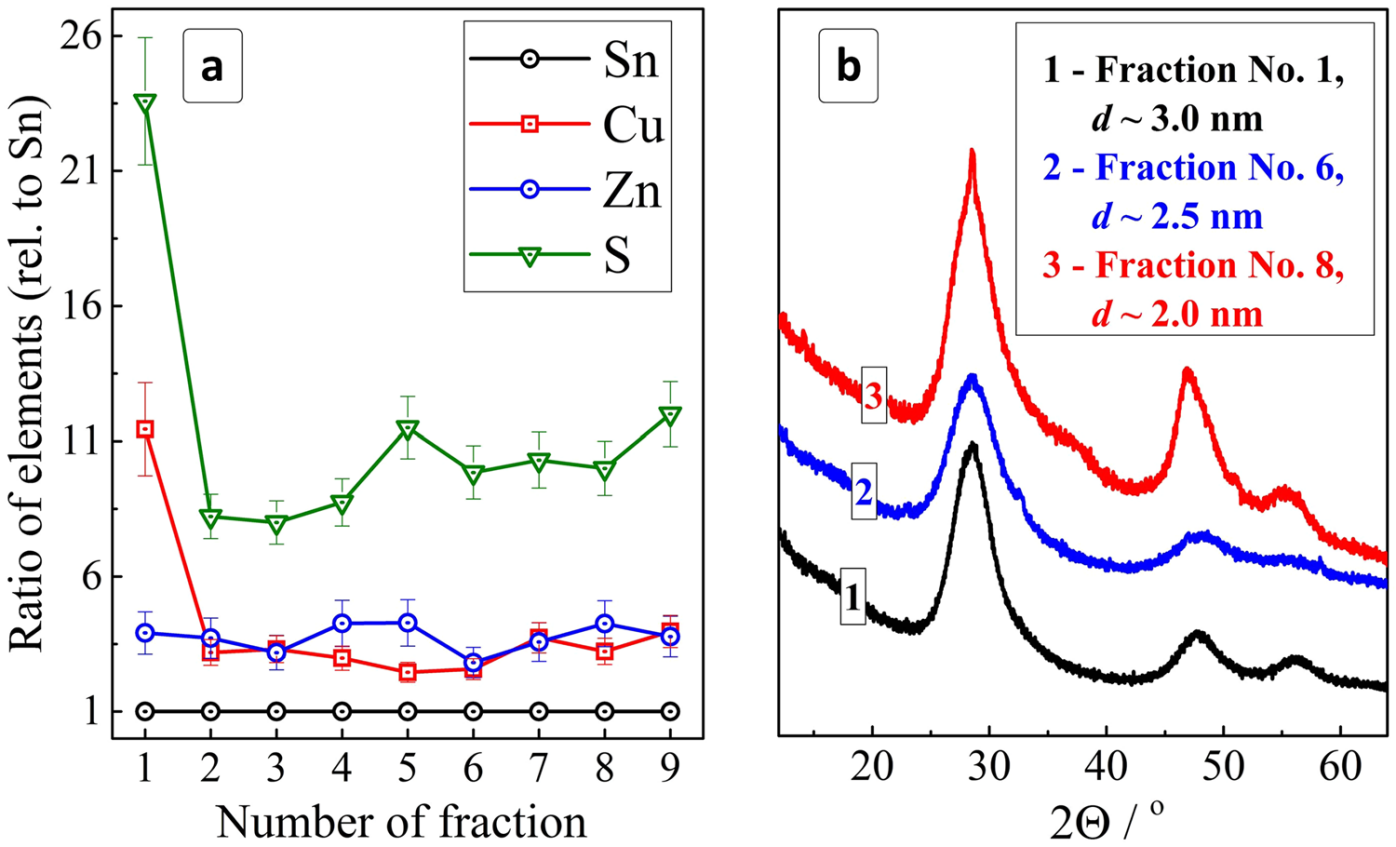
(**a**) Atomic ratios of elements (normalized to Sn) derived from the XPS data for different fractions of the size-selected CZTS NCs. (**b**) XRD patterns of CZTS NCs from fractions 2, 5, and 8 (initial metal ratio is Cu:Zn:Sn = 2:1:1).

An XPS study of the size-selected CZTS NCs showed the first fraction to be strongly enriched with copper and sulfur (Fig. 3a). We assume that this fraction contains the largest NCs from the original ensemble as well as an excess of copper-MA complexes remaining adsorbed on the NC surface as discussed above when interpreting the XPS data taken from the as-prepared CZNS NC ensemble. Starting from fraction No. 2 the samples show reasonable and rather constant Cu:Sn and Zn:Sn ratios of around (3–4):1. The contribution of sulfur increases somewhat with an increase of the fraction number, most probably reflecting an increase of the surface-to-volume ratio with the decrease of the NC size and a corresponding increase of the relative amount of the surface-adsorbed MA anions per NC.

The size-selected CZTS NC fractions showed basically the same kesterite motif in the X-ray diffractograms as the initial ink (Fig. 3b), with the peak FWHMs visibly growing with increasing fraction number. Estimations using the Scherrer equation showed that the average size of the CZTS NCs decreases from ~3 nm for fraction No. 2 to ~2.5 nm for fraction No. 6 and to ~2 nm for fraction No. 8. Summarizing, the complex of XPS and XRD data discussed show that the size selection yields a series of colloidal CZTS NCs with very similar composition but a different average size varying from 2 to 3 nm, similar to the earlier observation on the MA-capped Ag-In-S and Ag-In-S/ZnS NCs^57,58^.

**Figure 4:**
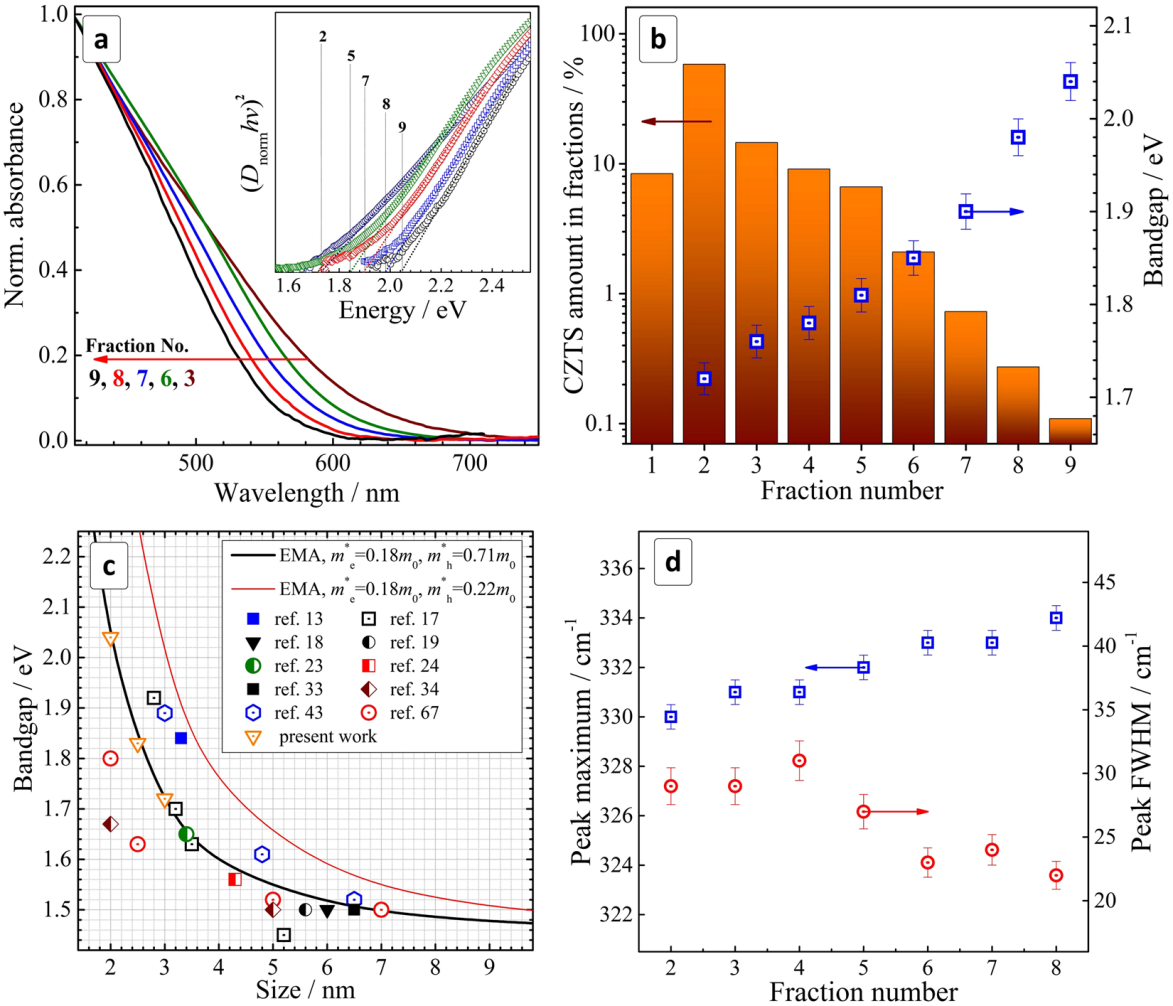
(**a**) Normalized absorption spectra of fractionated CZTS NCs (normalization at 420 nm). Inset: absorption spectra in the coordinates of the Tauc equation for direct electronic transitions. (**b**) Amount of CZTS NCs (bars, presented in % of the total CZTS amount) and NC bandgap (square with dot) in different fractions. (**c**) Relationship between *E*_g_ and size of CZTS NCs. Solid lines represent the results of calculations in the frame of the effective mass approximation using different sets of m^*^_e_ and m^*^_h_, scattered dots represent reported experimental results. (**d**) Position and FWHM of the main A_1_ Raman mode of CZTS NCs as a function of the fraction number.

The size-selected CZTS NCs show a continuously increasing absorption with a reasonably steep edge observed at ~650 nm for the largest NCs from fractions No. 2–3 (Fig. 4a). To evaluate the relative amount of CZTS in different fractions we adopted the optical density of fractionated colloids far from the band edge (at 420 nm) as a qualitative measure of the CZTS concentration. Apparently, this approach is approximate because the absorption cross section and even the type of electronic transition may be subject to changes with the NC size.

Nevertheless, this approximation provides a good visualization of the CZTS amount in different fractions (Fig. 4b). The chart shows that a relative loss associated with the discard of fraction no. 1 is not critically high. The largest number of particles is precipitated in fraction No. 2 displaying an average size of 3 nm, in accordance with the TEM data showing the peak of the size distribution at roughly the same size. The amount of CZTS in the following fractions decreases nearly exponentially and the fraction No. 9 has a by two orders of magnitude smaller concentration of CZTS NCs as compared to the most populated fraction No. 2.

As the size of the CZTS NCs decreases (with an increase in the fraction number) the absorption edge of the colloidal solutions display a “blue” shift reaching 550–560 nm for the smallest NCs from fraction No. 9. The spectra can be presented in the Tauc coordinates for direct interband electronic transitions (Fig. 4a, insert) showing extended linear sections wide enough for an accurate determination of the bandgaps (with an error of ±0.02 eV). We found that the bandgap *E*_g_ of the size-selected CZTS NCs increases steadily from 1.72 eV for fraction No. 2 to 2.04 eV for fraction No. 9 (Fig. 4b, blue rectangles), being in each case considerably larger than the bandgap of bulk kesterite CZTS (1.45–1.5 eV^51,74,75^). This indicates the strong spatial confinement of the charge carriers in the size-selected NCs resulting in the increase of their kinetic energies and their bandgaps *E*_g_.

It was earlier reported that a size-dependence of the absorption is observed for CZTS NCs smaller than 4–5 nm and, therefore, a strong size-dependence of the bandgap is expected for the present 2–3 nm CZTS NCs. We collected the reported data on the size-dependent bandgaps of different CZTS NCs to assess if these data can be reasonably described by the effective mass approximation model as it applies quite well for many II-VI semiconductor NCs^76^.

The effective-mass-approximation-based calibration curve was calculated using the well-known *E*_g_(*R*) dependence, where two minor terms corresponding to the Coulomb electron-hole interaction and Rydberg energy were neglected^76^:

$${\rm{\Delta }}{E}_{g}={(\pi {\hbar })}^{2}{(2R)}^{-2}(1/{m}_{e}^{\ast }+1/{m}_{h}^{\ast }).$$

Here, Δ*E*_g_ is a difference between the band gap of nanocrystalline and bulk semiconductor, m^*^_e_ and m^*^_h_ are effective masses of the conduction band electron and valence band hole, respectively, *R* is the NC radius, *ħ* is the reduced Planck constant.

Figure 4c shows two calibration curves produced by using two reported values of the effective hole mass m^*^_h_ of 0.71*m*_0_^13,43^ (the transverse contribution, *m*_0_ is the electron rest mass) and 0.18*m*_0_ (the longitudinal contribution)^17,43,74^. Experimental data of the size-dependence of *E*_g_ for CZTS NCs are presented as scatter plots including the evaluations made from direct TEM measurements^17–19,23,24,33,34^ as well as from XRD data using the Scherrer equation^13,43,66^. The experimental data are obviously clustered around the calibration curve produced with the transverse m^*^_h_ contribution showing a good applicability of this curve for the evaluation of the CZTS NC size from the optical bandgap. In particular, the average size of CZTS NCs in the terminal fractions of No. 2 (*E*_g_ = 1.72 eV) and No. 9 (*E*_g_ = 2.04 eV) was evaluated as 3 nm and 2 nm, respectively (green dashed lines in Fig. 4c), which is in good agreement with the XRD results.

As the general size range of the reported CZTS NCs is rather narrow, from 2 to 3 nm, we could not derive reproducible and reliable size distributions in separate fractions from the TEM data. Alternatively, to evaluate the NC size distributions in fractions we used an approach of Pesika *et al*.^77,78^ developed for the size-selected ZnO NCs which is of a general character and can be applied to a broad range of direct-bandgap semiconductor NCs showing signs of the spatial exciton confinement. According to this approach, the NC radii *R* distribution *n*(*R*) can be estimated from the NC-volume-normalized first derivative of the absorption onset *n*(*R*) ~ (*dD*/*dR*) × (^4^/_3_π*R*^3^)^−1^, where *D* is the optical density of colloidal NC on a wavelength (energy) corresponding to a certain *R*^77,78^. Estimations performed for fractions #1,6,8 showed that all three fractions are characterized by a very similar size distribution of ±15% (ESI, Fig. S5), which is much narrower as compared to the starting NC ensemble.

Along with the size-dependence of the interband transition energy, phonon confinement was also reported for CZTS NCs observable as a size-dependent variation in the positions (and FWHM) of the characteristic vibrational modes^43^. These reports, however, are counterweighted by other work, where opposite trends^66^ or no size-dependence of the phonon spectra were observed^79^ showing this subject still to be quite controversial for CZTS NCs. To assess a possible size effect on the vibrational properties of the MA-capped size-selected CZTS NCs we registered their Raman spectra maintaining identical conditions (substrate, sample preparation history, and laser wavelength of 514.7 nm). The Raman spectra of all the size-selected samples studied are very similar in the structure and peak positions. The frequency of the main kesterite A_1_ mode (see the representative fit in Fig. 1c) was found to vary from 330 cm^−1^ for fraction No. 2 to 334 cm^−1^ for fraction No. 9 (Fig. 4d). The peak width was found to decrease from ~30 cm^−1^ for the first fractions to ~22 cm^−1^ for fraction No. 9 (Fig. 4d).

The increase of the phonon frequency with the reduction of NC size can be explained by the positive phonon dispersion featured for CZTS^80^. Narrowing of the phonon peak for smaller NCs may appear controversial at the first glance, as the stronger phonon confinement was usually reported to cause broadening of phonon peaks in Raman spectra^81^. However, the behavior of the FWHM of the phonon peaks for the size-selected series of the CZTS NCs could be well explained by the non-trivial phonon band structure of CZTS^80^ and by the narrowing NC size distribution in each next fraction (from 1 to 9).

## Conclusions

We characterized the structure, composition, and optical properties of colloidal mercaptoacetate-stabilized Cu_2_ZnSnS_4_ NCs produced by a “green” method directly in aqueous solutions in the form of stable and concentrated “inks” with a Cu_2_ZnSnS_4_ content of up to 33 g/L. A comparison of XPS and EDX data indicated that CZTS NCs are stabilized by surface MA complexes of copper and zinc.

A size-selective precipitation using 2-propanol as a non-solvent was found to yield a series of around ten fractions of CZTS NCs with an average size *d* varying from 3 nm to 2 nm and maintaining roughly the same composition. The size-dependent variation of the optical bandgap from 1.72 eV (*d* = 3 nm) to 2.04 eV (*d* = 2 nm) can be satisfactorily described in the frame of the effective mass approximation.

The size-selected CZTS NCs revealed a mild phonon confinement, the main phonon frequency (FWHM) varying from 330 cm^−1^ (30 cm^−1^) for *d* = 3 nm to 334 cm^−1^ (22 cm^−1^) for *d* = 2 nm. The trend complies with a positive phonon dispersion predicted earlier for CZTS^78^.

## Electronic supplementary material

Supplementary Information
