# Supplementary material for: “Green” Aqueous Synthesis and Advanced Spectral Characterization of Size-Selected Cu2ZnSnS4 Nanocrystal Inks
Source: Sci Rep. 2018 Sep 12;8:13677. doi: 10.1038/s41598-018-32004-1 (PMC6135749; doi:10.1038/s41598-018-32004-1)
Supplement: Supplementary file 1 — Supplementary Information [file 41598_2018_32004_MOESM1_ESM.doc]

***Electronic Supporting Information***

**“Green” Aqueous Synthesis and Advanced Spectral Characterization of Size-Selected Cu_2_ZnSnS_4_ Nanocrystal Inks**

**Oleksandr Stroyuk^a,b*^, Alexandra Raevskaya^a,b^,**

**Oleksandr Selyshchev^c^, Volodymyr Dzhagan^d^,**

**Nikolai Gaponik^a*^, Dietrich R.T. Zahn^c^, Alexander Eychmüller^a^**

***^a^****Physical Chemistry, TU Dresden, 01062 Dresden, Germany*

***^b^****L.V. Pysarzhevsky Institute of Physical Chemistry,*

*National Academy of Sciences of Ukraine, Kyiv, 03028, Ukraine*

***^c^****Semiconductor Physics, Chemnitz University of Technology, 09107 Chemnitz, Germany*

***^d^****V. E. Lashkaryov Institute of Semiconductors Physics,*

*National Academy of Sciences of Ukraine, Kyiv, 03028, Ukraine*

**Authors for correspondence**:

^*^***Dr. Oleksandr Stroyuk***, Physikalische Chemie, Technische Universität Dresden, Bergstraße 66b, 01062 Dresden, Germany, Tel. +49(0)351 463 34351; Laboratory of Organic Photovoltaics and Electrochemistry, L.V. Pysarzhevsky Institute of Physical Chemistry, National Academy of Sciences of Ukraine, Kyiv, Prosp. Nauky 31, 03028, Ukraine, Tel. +38(0) 44 525 02 70,

*e-mail*: alstroyuk@ukr.net; oleksandr.stroyuk@chemie.tu-dresden.de

^*^***Prof. Nikolai Gaponik***, Physikalische Chemie, Technische Universität Dresden, Bergstraße 66b, 01062 Dresden, Germany, Tel. +49(0)351 463 35203, Fax: +49(0)351 463 37164,

*e-mail*: nikolai.gaponik@chemie.tu-dresden.de


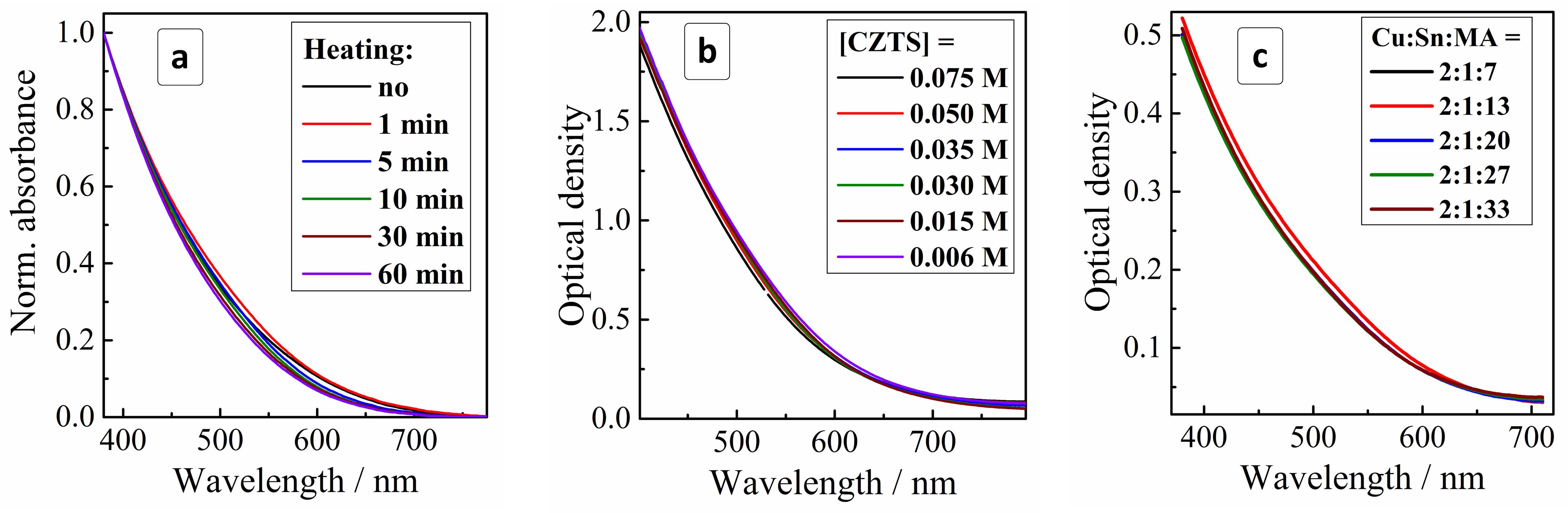


**Figure S1**. (a) Normalized absorption spectra of CZTS NCs produced at different durations of heating. (b) Absorption spectra of CZTS NCs synthesized at a different concentrations of precursors (after dilution to the same concentration). (c) Absorption spectra of colloidal CZTS NCs synthesized at a different MA content. Solutions diluted by a factor of 100 for the measurements.


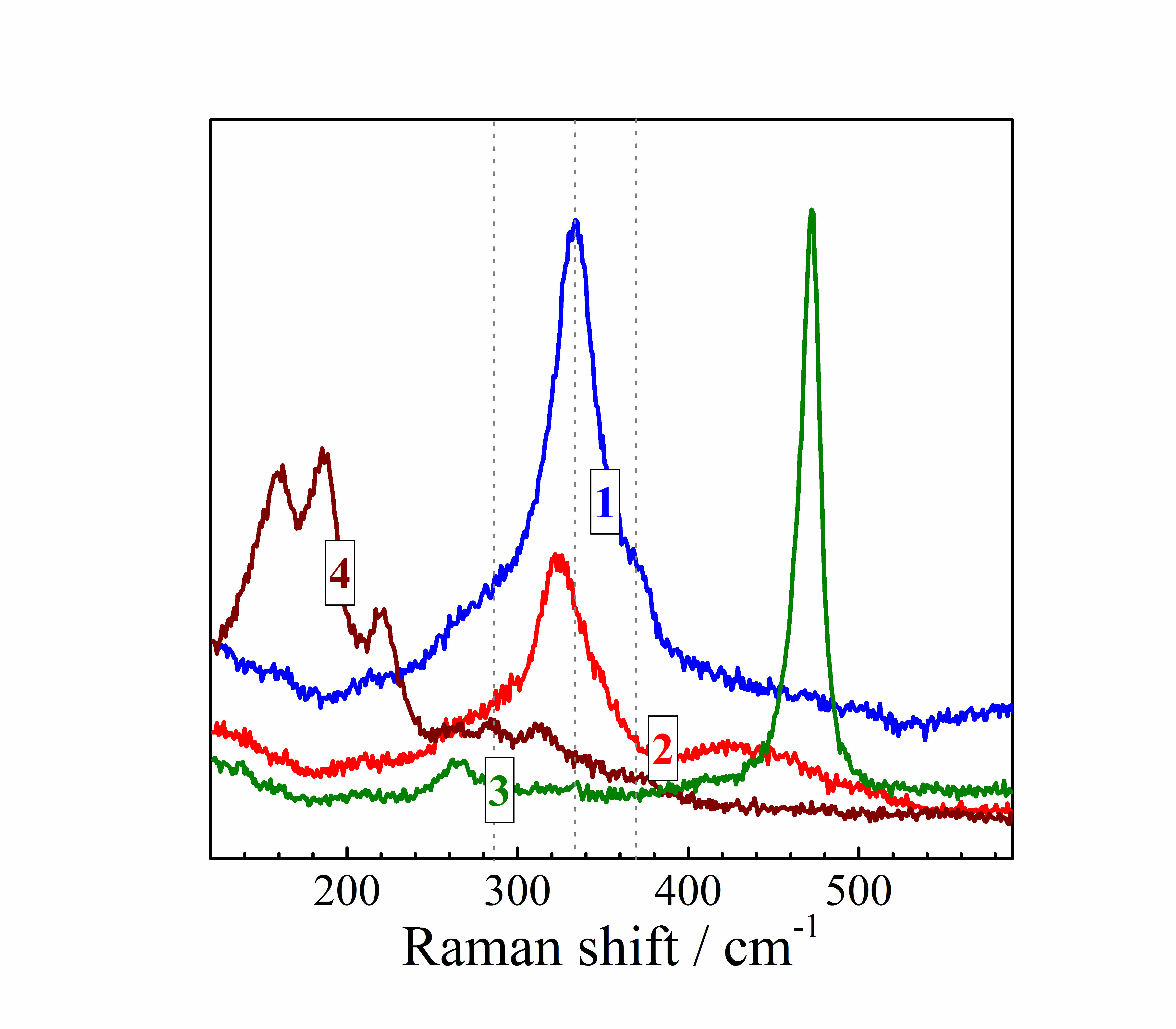


**Figure S2**. Raman spectra of CZTS NCs (curve 1) as well as Cu-Sn-S (2), Cu-S (3), and Sn-S (4) samples produced under identical conditions.

**
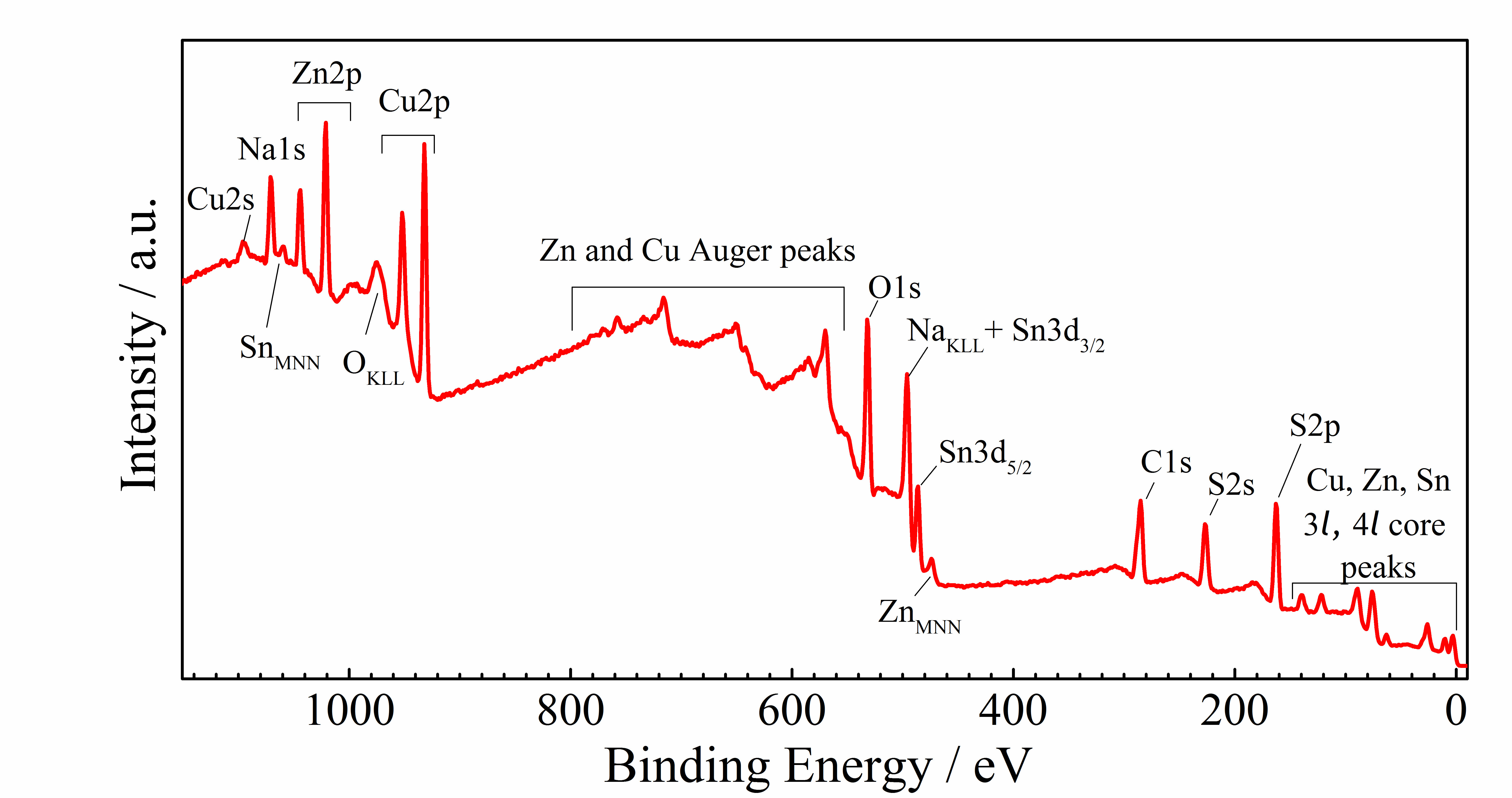
**

**Fig. S3**. Survey X-ray photoelectron spectrum of an unfractionated MA-stabilized CZTS colloid containing an excess of sodium mercaptoacetate.


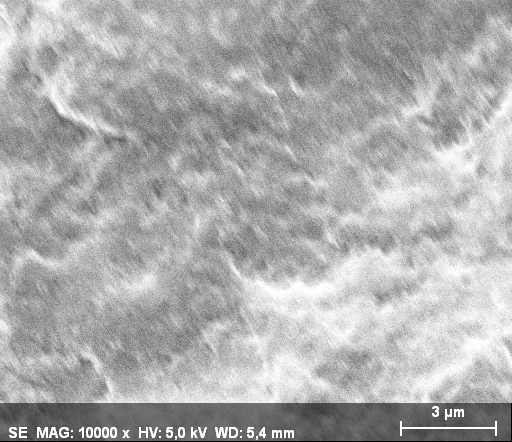

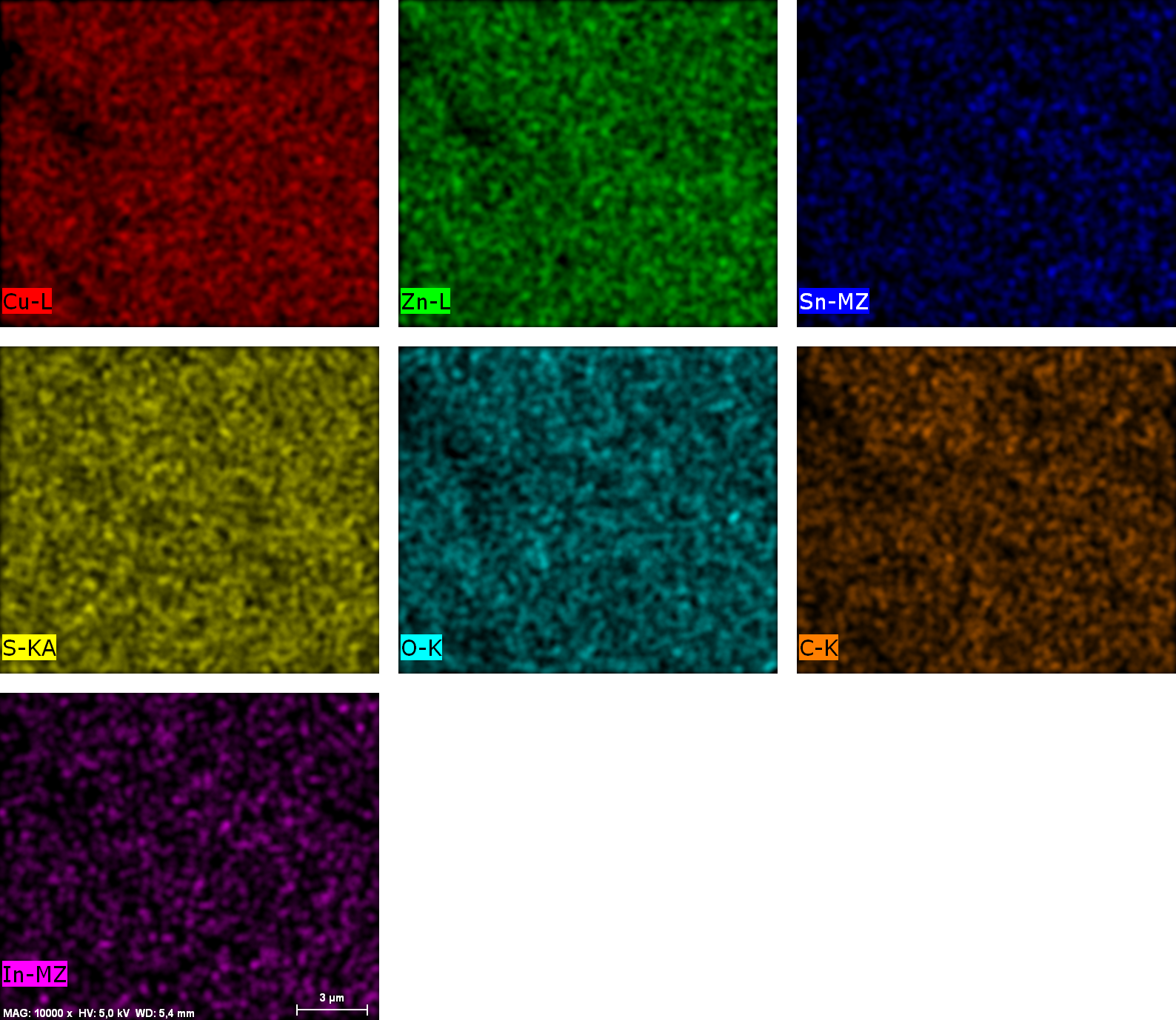


a b

**Fig. S4**. SEM (a) and EDX element maps (b) of atoms in the CZTS/ITO sample.

**Table S1**. Composition of unfractionated CZTS NCs produced at a molar Cu:Zn:Sn ratio of 2:1:1 as determined by EDX.

| Element | Weight % | Atomic % | Error in at.% |
| --- | --- | --- | --- |
| C | 6 | 23 | 2 |
| O | 6 | 18 | 2 |
| S | 11 | 16 | 2 |
| Cu | 27 | **20** | 5 |
| Zn | 15 | **10** | 3 |
| Sn | 36 | **14** | 12 |

+


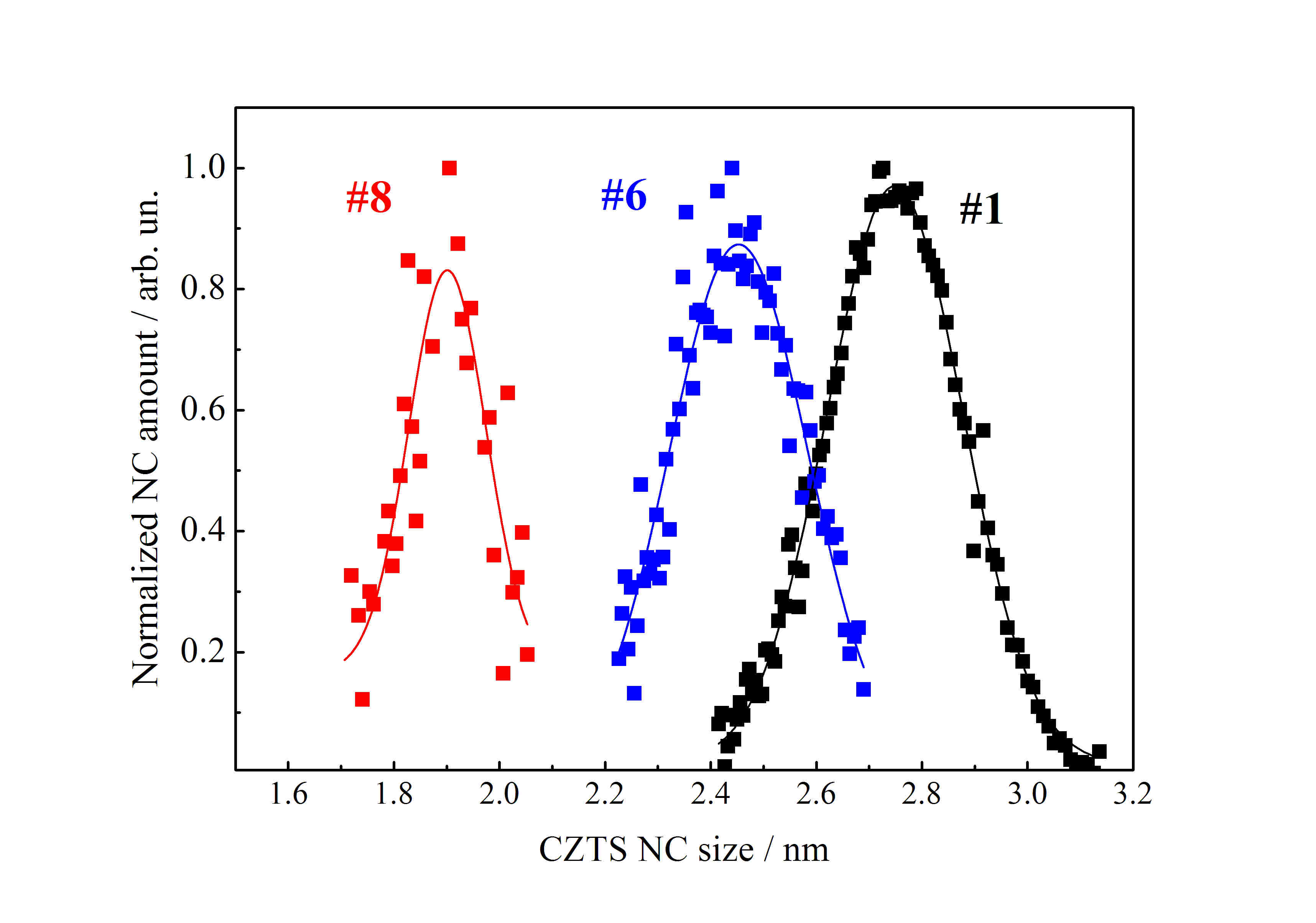


**Fig. S5**. Size distributions of CZTS NCs in fractions #1, #6 and #8 calculated from the corresponding absorption onsets.
